# Work participation of patients with musculoskeletal disorders: is this addressed in physical therapy practice?

**DOI:** 10.1186/s12995-017-0174-5

**Published:** 2017-08-25

**Authors:** Wiebke Oswald, Nathan Hutting, Josephine A. Engels, J. Bart Staal, Maria W. G. Nijhuis-van der Sanden, Yvonne F. Heerkens

**Affiliations:** 1 Faculty of Health and Social Studies, Research Group Occupation & Health, HAN University of Applied Sciences, P.O. Box 6960, 6503 GL Nijmegen, Netherlands; 2 Faculty of Health and Social Studies, Physical Therapy, HAN University of Applied Sciences, Nijmegen, Netherlands; 3 Faculty of Health and Social Studies, Research Group Musculoskeletal Rehabilitation, HAN University of Applied Sciences, Nijmegen, Netherlands; 4 Radboud Institute for Health Sciences, IQ Healthcare, Radboud University Medical Center, Nijmegen, Netherlands

**Keywords:** Musculoskeletal disorders, Physical therapy, Occupational health, Work participation

## Abstract

**Background:**

Musculoskeletal disorders are the main complaints for visiting a physical therapist (PT) in primary health care; they have a negative effect on an individual’s quality of life and result in a major cost to society. Qualitative research has shown that physical therapists (PTs) treating patients with these disorders experience barriers in the integration of occupational factors within their practice, and also revealed a lack of cooperation between PTs and (other) occupational healthcare providers. The aim of this study is to quantitatively investigate how generalist PTs in the Netherlands, who treat patients with musculoskeletal disorders, currently integrate occupational factors within their practice, and to identify their opinions and needs with regard to enhancing the integration of the patient’s work within physical therapy practice.

**Methods:**

A cross-sectional survey was conducted among generalist PTs who treat working-age (18–67 years) patients with musculoskeletal disorders. Generalist PTs were contacted for participation via digital news-mails and asked to fill out an online survey which was developed based on the results of a recent qualitative study. The survey consisted of: i) demographics of the participants, ii) questions on how generalist PTs currently integrate occupational factors within their practice, and iii) asked their opinion about the integration of occupational factors within physical therapy. The PTs were also asked about their needs with regard to the integration of occupational factors and with regard to cooperation with other (occupational) health professionals. All answers (using Likert scales) are presented as the *number* and *percentage* of the respondents reporting those specific answers, whereas all other answers are presented as means and standard deviations.

**Results:**

Of the 142 respondents, 64% indicated that occupational factors should be addressed to a greater extent within physical therapy. To have the possibility to bill for a workplace assessment (60.6%) and more knowledge about laws and regulations (50%) were identified as needs of the respondents. Only 14.8% of the respondents indicated that they communicate with or consult a PT specialized in occupational health. Only 12.7% of the participants who do not have a specialized PT within their practice sometimes/regularly refer patients to a specialized PT.

**Conclusions:**

Although generalist PTs address occupational factors within their practice, there is room for improvement. This study also identified a lack of cooperation between generalist PTs and PTs specialized in occupational health.

**Electronic supplementary material:**

The online version of this article (10.1186/s12995-017-0174-5) contains supplementary material, which is available to authorized users.

## Background

The main complaints for visiting a physical therapist (PT) in primary healthcare worldwide are musculoskeletal disorders (MSDs) [1]. MSDs have a negative effect on an individual’s quality of life and are a major cost to society [2, 3]. The most common MSDs (e.g. back and neck/shoulder problems) are the main cause of disability among the working-age (18–67 years) population [2]. In Great Britain in 2013–2014, the prevalence of work-related MSDs was 1680 per 100,000 employees, and the incidence of new cases was 550 per 100,000 employees [4].

Work-related MSDs are disorders whereby work activities and conditions significantly contribute to the onset or progression of the disorder, but are not necessarily the sole cause of the disorder [5]. In the development of MSDs, in addition to other risk factors, the work environment and its characteristics can play an important role. For example, adverse working conditions, psychosocial problems, lack of proper attention to ergonomics, postural problems during work, and mental overload/underload [5]. Even though it remains unclear whether work exposure is responsible for the onset or progression of MSDs, work-related MSDs play a considerable role in the financial burden on society, which continues to increase [6–8].

Although work exposure can cause health problems, work also has a beneficial influence on the individual’s wellbeing [9]. Moreover, work participation is an important factor of perceived quality of life [10, 11]. Long periods of absenteeism lead to a smaller chance of return to work and an increased risk of problems related to physical, mental and social wellbeing [11, 12]. Research has shown that return to work can be an important component of rehabilitation [13]. For example, if interventions are provided within a few weeks after the complaints started, or in the early stage of absenteeism, they can contribute to the prevention of long-lasting absenteeism [13–16].

In the Netherlands, primary healthcare and occupational healthcare are two separate systems. Dutch patients with work-related MSDs often consult health professionals in primary healthcare rather than in occupational healthcare. Although participating in work is an important factor of wellbeing [10], most caregivers in primary healthcare -including physical therapists (PTs)- pay insufficient attention to the relation between MSDs and occupational factors among their working patients [17]. Absenteeism can be reduced by improving primary healthcare and by improving the communication between health professionals, occupational health professionals, the workplace, the patient and the insurer [18, 19]. Therefore, the Social and Economic Council of the Netherlands (SER) recommended to improve the quality of primary, secondary and tertiary healthcare, by increasing knowledge about and by focusing on prevention of absenteeism, return to work, and accessibility to occupational healthcare for all employees [17]. More attention, knowledge and collaboration in occupational-related healthcare could lead to a cost reduction of more than 1 billion euro a year [17, 20].

In 2016, almost 25% of the Dutch working-age population visited a PT in primary healthcare due to MSDs in the past year [21]. With their expertise on MSDs, complemented by knowledge and skills in managing occupational factors, PTs can play an important role in facilitating rehabilitation, return to work (RTW) and prevention of absenteeism [19, 22]. There is strong evidence in both the occupational health and physiotherapy literature to support the role of PTs as primary care providers for injured workers with MSDs [22]. Provision of treatments to address specific MSD pathology is integral to recovery, which can facilitate earlier RTW. The interventions that consistently demonstrate greater efficacy for positive work outcomes tend to be multi-modal and multi-disciplinary, including PTs [22]. Common modalities used by PTs include advice and education, manual therapy, and exercise [22]. One review found that i) patient education had some effect on RTW for workers with low back pain, ii) physical exercise was effective in subacute and chronic low back pain workers, and iii) graded activity had positive effects on RTW, pain and functional status [23]. However, studies in the UK show that primary healthcare providers, including PTs, do not provide guidance and support to workers with regard to their work problems [24, 25].

In a recent qualitative study performed by our group in the Netherlands [26], PTs stated that they experience barriers in the integration of occupational factors in their practice. The Netherlands has an educational program in which generalist PTs can specialize in occupational physical therapy or work-related physical therapy; nevertheless, less than 1% of the PTs is registered as an occupational physical therapist (OPT) or work-related physical therapist (WPT). The OPT is specialized in creating a healthy work environment and reducing sick leave within an organization, whereas the WPT is specialized in enhancing the capacity for recovery of employees with occupational health complaints. Work-related PTs are geared towards the reintegration of employees through individual and work-orientated exercises and treatment; they treat employees in a private practice setting or (if necessary and/or relevant) within the organization itself [27]. Although generalist PTs treat working-age patients with MSDs, they may hesitate to include occupational factors in their treatment, irrespective of whether or not they found work an important factor. Furthermore, PTs do not always cooperate with occupational physical therapists (OPTs) in order to provide sufficient care to their working-age patients [26]. Based on the results of the qualitative study [26] it was concluded that a quantitative study is needed to investigate (on a broader scale) how generalist PTs in the Netherlands integrate their patients’ occupational factors within the physical therapy, and which barriers and facilitators influence the integration.

Therefore, this study aims to i) quantitatively investigate how PTs is the Netherlands, who treat patients with MSDs, currently integrate occupational factors within their practice, and ii) identify their opinions and needs with regard to enhancing the integration of the patient’s work within physical therapy.

## Methods

### Procedures and participants

A cross-sectional survey was conducted among PTs who treat working-age patients with MSDs. PTs in the Netherlands were contacted (during November–December 2016) for participation via calls in digital news-mails of the Royal Dutch Society for Physical Therapy (KNGF), the Dutch Institute of Allied Health Professions (NPi), and social media networks of the researchers (LinkedIn). Included for this survey were generalist PTs who treat working-age patients with MSDs, whereas OPTs and work-related physical therapists (WPTs) were excluded in order to get a clear view of the opinions of generalist PTs who have not specialized in occupational healthcare. The call in the news-mails explained the purpose of the study and the voluntary nature of participation. The PTs were asked to fill out an online survey, provided via Parantion Easion Survey version 3.63. A link to log on to the website anonymously was included in the digital newsletter. To encourage participation, all PTs who filled in the survey had the option to fill in their e-mail address (voluntary) in order to win a gift card of 60 euro.

### Survey

Based on the results of our recent qualitative study [26], a survey was developed for the present investigation. The qualitative study investigated how PTs integrate occupational factors within their practice, what they need for a better cooperation with other occupational health professionals, and which factors can facilitate a better integration of occupational factors within physical therapy [26]. An overview of the main categories and underlying themes identified in the earlier qualitative study are presented in Additional file 1: Appendix 1. The survey was pretested by two generalist PTs and reviewed by the members of the research group. No major modifications of the survey were found to be necessary.

The first part of the survey assessed demographics of the participating PTs in terms of gender, age, years of experience as PT, and PTs’ specialization. With regard to specialization and referral to occupational health professionals, it was possible to choose more than one specialization. The survey consisted of questions on how PTs currently integrate occupational factors within their practice, and on their opinion on the integration of occupational factors within physical therapy. The PTs were also asked about their needs with regard to the integration of occupational factors (need of knowledge, skills and tools) and with regard to cooperation with WPTs, OPTs, and other occupational health professionals.

Part of the survey was based on the concepts awareness, attitude, social influences, and self-efficacy of the I-Change model [28]. Awareness of the importance of involving occupational factors in their practice was investigated (Table 2, question 1a–d). Attitude was assessed by asking the PTs whether they had a positive (Table 2, question 2a-e, 2k) or a negative attitude (Table 2, question 2f-g, 2i-j) towards involving occupational factors in their practice. Social influences were assessed in terms of norms, modeling, support, and pressure (Table 2, question 3a–e). Furthermore, self-efficacy was assessed (Table 2, question 4a–f). For all questions, unless stated otherwise, a 5-point Likert scale was used for response, ranging from strongly disagree to strongly agree, or ranging from never to always.

### Data analysis

Demographics are presented as means and standard deviations (SDs), while gender and specialization are presented as number and percentage. All answers using Likert scales are presented as the *number* and the *percentage* of the respondents reporting that specific answer, whereas all other answers are presented as means and standard deviations (SDs).

The Statistical Package for the Social Science (SPSS, version 22) was used to analyze the data.

## Results

In this study, a total of 142 PTs completed the survey. The mean age of the PTs was 42.4 (SD 13.3) years, 49.3% were male and 50.7% were female. The average years of work experience was 18.4 (SD 13.0) years. The most common postgraduate specializations were manual therapy (38.7%) and sports physical therapy (14.1%). Psychosomatic physical therapy was reported as a specialization by 8.5% of the PTs, and edema physical therapy was reported by 5.6%. A minority of the PTs were specialized in orofacial physical therapy (2.1%), haptonomy (1.4%), and pelvic physical therapy (0.7%). The category ‘other’ was chosen by 40.8% of the PTs. The participating PTs estimated that 67.6% (SD 18.2) of their patients were of working age and that 66.9% (SD 20.5) of their patients had paid work.

Table 1 describes to what extent PTs address occupational factors of patients with paid work within the patient interview, pay attention to their patients work/work activities during the phases of physical therapy, give advice about occupational factors during treatment, and perform actions to investigate the workplace of their patients. Table 1 also describes in what situations PTs refer their patients to an occupational health professional.

**Table 1:** Number and percentage of generalist physical therapists (*n* = 142) addressing several occupational factors within physical therapy practice

	Never, n (%)	Rarely, n (%)	Sometimes, n (%)	Regularly, n (%)	Always, n (%)
1. To what extent do you address the following items within the patient interview in patients with paid work?					
a) Load and capacity in relation to work activities	0	1 (0.7)	7 (4.9)	36 (25.4)	98 (69.0)
b) Work tasks/work operations of the patient	0	1 (0.7)	14 (9.9)	56 (39.4)	71 (50.0)
c) Work method/work technique of the patient	0	10 (7.0)	35 (24.6)	49 (34.5)	48 (33.8)
d) Workplace of the patient	1 (0.7)	8 (5.6)	36 (24.5)	54 (38.0)	43 (30.3)
e) Working hours of the patient	2 (1.4)	20 (14.1)	37 (26.1)	49 (34.5)	34 (23.9)
f) Work pressure of the patient	0	7 (4.9)	24 (16.9)	60 (42.3)	51 (35.9)
g) Workplace absenteeism	1 (0.7)	20 (14.1)	32 (22.5)	42 (29.6)	47 (33.1)
h) Working together with supervisor/colleagues	8 (5.6)	38 (26.8)	51 (35.9)	25 (17.6)	20 (14.1)
i) Return to work (in case of absenteeism)	1 (0.7)	5 (3.5)	21 (14.8)	67 (47.2)	48 (33.8)
j) Importance of work participation for the patient	1 (0.7)	3 (2.1)	32 (22.5)	61 (43.0)	45 (31.7)
k) Degree to which the patient thinks that there is a relationship between the complaints and the patient’s work	2 (1.4)	5 (3.5)	23 (16.2)	65 (45.8)	47 (33.1)
l) Recovery options within the work situation	1 (0.7)	3 (2.1)	32 (22.5)	61 (43.0)	45 (31.7)
m) Recovery options outside the work situation	2 (1.4)	5 (3.5)	23 (16.2)	65 (45.8)	47 (33.1)
2. To what extent do you address the patient’s work/work activities during the following phases of practice?					
a) Physical examination	3 (2.1)	13 (9.2)	38 (26.8)	45 (31.7)	43 (30.3)
b) Treatment goals/treatment plan	1 (0.7)	4 (2.8)	12 (8.5)	60 (42.3)	65 (45.8)
c) Treatment	2 (1.4)	2 (1.4)	15 (10.6)	79 (55.6)	44 (31.0)
d) Evaluation	1 (0.7)	2 (1.4)	16 (11.3)	61 (43.0)	62 (43.7)
3. To what extent do you advise/give information (to) the patient during treatment about the following items?					
a) Load and capacity in relation to work activities	1 (0.7)	0	9 (6.3)	54 (38.0)	78 (54.9)
b) Work tasks/work operations of the patient	1 (0.7)	2 (1.4)	28 (19.7)	67 (47.2)	44 (31.0)
c) Work method/work technique of the patient	1 (0.7)	6 (4.2)	32 (22.5)	56 (39.4)	47 (33.1)
d) Workplace of the patient	3 (2.1)	7 (4.9)	33 (23.2)	70 (49.3)	29 (20.4)
e) Working hours of the patient	7 (4.9)	32 (22.5)	52 (36.6)	35 (24.6)	16 (11.3)
f) Work pressure of the patient	2 (1.4)	10 (7.0)	37 (26.1)	63 (44.4)	30 (21.1)
g) Workplace absenteeism	3 (2.1)	24 (16.9)	44 (31.7)	45 (31.7)	26 (18.3)
h) Working together with supervisor/colleagues	9 (6.3)	40 (28.2)	46 (32.4)	32 (22.5)	15 (10.6)
i) Return to work (in case of absenteeism)	1 (0.7)	3 (2.1)	29 (20.4)	69 (48.6)	40 (28.2)
j) Importance of work participation for the patient	1 (0.7)	5 (3.5)	31 (21.8)	71 (50.0)	34 (23.9)
k) Degree to which the patient thinks that there is a relationship between the complaints and the patient’s work	1 (0.7)	5 (3.5)	31 (21.8)	66 (46.5)	39 (27.5)
4. To what extent do you perform the following actions during treatment?					
a) Let the patient imitate work activities	4 (2.8)	14 (9.9)	44 (31.0)	67 (47.2)	13 (9.2)
b) Let the patient make photos/videos of the workplace	62 (43.7)	38 (26.8)	26 (18.3)	14 (9.9)	2 (1.4)
c) Visit the patient’s workplace	80 (56.3)	40 (28.2)	16 (11.3)	6 (4.2)	0
d) Use questionnaires or screening lists about work or aspects of work	50 (35.2)	46 (32.4)	23 (16.2)	20 (14.1)	3 (2.1)
5. When I refer patients to a professional in the field of occupational health it is:					
a) Directly after the history taking	45 (31.7)	51 (35.9)	36 (25.4)	9 (6.3)	1 (0.7)
b) In case of disappointing recovery	19 (13.4)	18 (12.7)	64 (45.1)	32 (22.5)	9 (6.3)
c) In case of failure of recovery	18 (12.7)	16 (11.3)	49 (34.5)	37 (26.1)	22 (15.5)
d) In case of recurrent complaints	16 (11.3)	28 (19.7)	71 (50.0)	23 (16.2)	2 (2.8)

The participating PTs stated that, if they communicate with or consult other occupational health professionals, then they mainly have contact with occupational health/insurance physicians (72.5%; in the Netherlands, insurance physicians assess the disability claims of injured workers) and occupational therapists (31.7%). A minority of the PTs has contact with employers (17.6%), WPTs (14.8%), Human Resource managers (12%), OPTs (11.3%) and labor experts (10.6%).

The majority of the PTs (76.8%) had no direct OPT or WPT colleague. Of this 76.8%, the majority (60%) never refer to a WPT outside their practice/company, 27.3% rarely refer to a WPT, 10% sometimes refer to a WPT, and 2.7% regularly refer their patients to a WPT outside their practice. Of the generalist PTs who have a WPT as colleague, 6.1% never and 18.2% rarely work together. About 64% of the generalist PTs do not want to have more intensive cooperation with a WPT, whereas 53.5% would like to have more contact with OPTs within the patient’s company. Also, 54.9% of the PTs would like to have more intensive cooperation with occupational health and insurance physicians.

Table 2 describes the proportion of the opinions of PTs with regard to addressing occupational factors within physical therapy in terms of awareness, attitude, social influences, and self-efficacy. Table 3 presents the needs of PTs with regard to the involvement of occupational factors within physical therapy.

**Table 2:** Number and percentage of the opinions of generalist physical therapists (*n* = 142) with regard to addressing occupational factors within physical therapy practice in terms of awareness, attitude, social influences and self-efficacy

	Totally disagree, n (%)	Disagree, n (%)	Neutral, n (%)	Agree, n (%)	Totally agree, n (%)
1. Awareness *Instruction: Please indicate to what extent you agree or disagree with the following statements:*					
a) The border where general physical therapy ends and occupational health physical therapy starts is clear to me.	10 (7.0)	64 (45.1)	38 (26.8)	27 (19.0)	3 (2.1)
b) I am aware of the fact that, on average, the majority of my patients have paid work.	5 (3.5)	8 (5.6)	15 (10.6)	61 (43.0)	53 (37.3)
c) I see the way my patients work (performance) as a possible treatment option.	3 (2.1)	4 (2.8)	21 (14.8)	78 (54.9)	36 (25.4)
d) I see the extent of work participation of my patient as a possible treatment option.	4 (2.8)	4 (2.8)	24 (16.9)	81 (57.0)	29 (20.4)
2. Attitude *Instruction: The following statements are about your opinion regarding physical therapy in general, not just about your own actions.*					
a) The patient’s work should have a larger place within the physical therapy than it currently does	2 (1.4)	12 (8.5)	37 (26.1)	66 (46.5)	25 (17.6)
b) I consider the patient’s work as a major barrier or facilitator for recovery.	3 (2.1)	5 (3.5)	14 (9.9)	81 (57.0)	39 (27.5)
c) I consider the patient’s work as a major cause of complaints.	4 (2.8)	13 (9.2)	(46 (32.4)	58 (40.8)	21 (14.8)
d) Addressing the patient’s work in practice has positive effects on the patient’s performance.	1 (0.7)	0	14 (9.9)	82 (57.7)	45 (31.7)
e) Addressing the patient’s work in physical therapy practice has a positive effect on the patient’s work absenteeism.	2 (1.4)	4 (2.8)	31 (21.8)	73 (51.4)	32 (22.5)
f) It is not important to address the patient’s work/work activities within physical therapy practice because the patients visit the physical therapist for treating his/her physical complaints.	87 (61.3)	39 (27.5)	5 (3.5)	8 (5.6)	3 (2.1)
g) There is insufficient opportunity to address the patient’s work/work activities within the regular physical therapy treatment.	51 (35.9)	59 (41.5)	5 (10.6)	16 (11.3)	1 (0.7)
h) Cooperation with the business community about occupational health offers a possibility to put more focus on physical therapy and create a different business model.	4 (2.8)	4 (2.8)	31 (21.8)	68 (47.9)	35 (24.6)
i) The domain ‘work’ belongs to the company/occupational health physical therapist, whereas generalist physical therapists have little to do with it.	47 (33.1)	63 (44.4)	21 (14.8)	8 (5.6)	3 (2.1)
j) The specific knowledge that is required in the field of occupational health is not sufficiently accessible to the generalist physical therapist.	17 (12.0)	31 (21.8)	55 (38.7)	34 (23.9)	5 (3.5)
k) The patient’s demand of care is rarely related to his/her work.	44 (31.0)	58 (40.8)	23 (16.2)	14 (9.9)	3 (2.1)
3. Social influences *Instruction: The following statements relate to your personal situation and about your personal opinion. Please indicate to what extent you agree or disagree with the following statements:*					
a) My colleagues think that I should not address the patient’s work in my practice.	69 (48.6)	49 (34.5)	20 (4.1)	4 (2.8)	0
b) The majority of my colleagues pay sufficient attention to their patient’s work in their practice.	5 (3.5)	30 (21.1)	47 (33.1)	53 (37.3)	7 (4.9)
c) I notice that patients like it when I discuss their work situation with them.	3 (2.1)	6 (4.2)	21 (14.8)	88 (62.0)	24 (16.9)
d) My colleagues support me to pay attention to the work of my patient.	6 (4.2)	15 (10.6)	62 (43.7)	50 (35.2)	9 (6.3)
e) My supervisor supports me to pay attention to the work situation of my patient.	6 (4.2)	12 (8.5)	69 (48.6)	40 (28.2)	15 (10.6)
4. Self-efficacy *Instruction: Please indicate to what extent you agree or disagree with the following statements:*					
a) I am able to address the work of my patient within my practice, despite the ever-changing laws and regulations in this area.	3 (2.1)	12 (8.5)	38 (26.8)	75 (52.8)	14 (9.9)
b) I am able to ask my patient about his/her working situation and to adapt my treatment accordingly.	1 (0.7)	3 (2.1)	16 (11.3)	96 (67.6)	26 (18.3)
c) Based on my current knowledge, I am optimally able to treat patients who work and to advise them with regard to work tasks/work technique, workplace working hours, workload, and work absenteeism.	6 (4.2)	34 (23.0)	43 (30.3)	44 (31.0)	15 (10.6)
d) I find it difficult to assess to what extent the patient’s complaint is work related.	14 (9.9)	61 (43.0)	29 (20.4)	36 (25.4)	2 (1.4)
e) I am able to hold a substantive discussion about the patient with an occupational health physician or occupational health physical therapist.	2 (1.4)	14 (9.9)	28 (19.7)	71 (50.0)	27 (19.0)
f) Based on my knowledge of the field of occupational therapy, I am able to refer patients to the occupational health physical therapist if indicated.	9 (6.3)	29 (20.4)	38 (26.8)	45 (31.7)	21 (14.8)

**Table 3:** Overview of the needs of physical therapists with regard to the involvement of occupational factors within physical therapy practice

	n (%)
To have the possibility to bill for a workplace assessment.	86 (60.6)
Questionnaires about the work participation of the patient.	75 (52.8)
Screening lists to assess to what extent the patient’s complaint is work related.	74 (52.1)
More knowledge about occupational health-related laws and regulations.	71 (50.0)
More knowledge about the domain and the position of the occupational health physical therapist.	61 (43.0)
More knowledge about the domain and position of occupational health professionals such as the occupational health nurse, occupational health physician, insurance physician, medical consultant and human resources consultant.	58 (40.8)
More attention to the factor work in the basic physical therapy educational programs.	56 (39.4)
More attention to work within the physical therapy guidelines.	55 (38.7)
More knowledge about work tasks/work activities, work methods/technique, workplace, working hours, workload.	52 (36.6)
More time in the treatment session to discuss work-related factors.	52 (36.6)
More practical tools to integrate work within my practice.	51 (35.9)
More practical skills to carry out a basic workplace assessment.	51 (35.9)
More opportunities to give ‘work’ a proper place within the electronic patient’s file.	40 (28.2)
More attention to the factor work in the Master educational programs for physical therapy specializations (if applicable).	25 (17.6)

## Discussion

This study investigated to what extent generalist PTs in the Netherlands currently integrate occupational factors in their treatment of patients with MSDs within primary care and evaluated their opinions/needs with regard to enhancing integration of the patient’s work within physical therapy.

### Importance of addressing occupational factors

Most respondents were aware of the importance of involving occupational factors within their care. Most of the respondents had a positive attitude towards paying (more) attention to occupational factors. They found it important to address the patient’s work because it has a positive effect on the patient’s performance. This is consistent with findings from our recent qualitative research [26]. In general, the respondents had high self-efficacy (i.e. beliefs about their capabilities to produce designated levels of performance) with regard to integrating occupational factors within their practice. With regard to social support, the perceived support of colleagues and supervisors of the respondents was not clearly shown in this study. However, with regard to the social support of patients, most respondents received appreciation with regard to involving occupational factors within their care.

### Addressing occupational factors in practice

In this study, most respondents stated that they integrate occupational factors of patients in their care. Generally, respondents indicated that they regularly or always address occupational factors within their patient interview. However, almost a quarter of the respondents indicated that they only sometimes pay attention to work methods, the workplace, and absenteeism of their patients. Respondents had a positive attitude and experienced awareness, social support, and high self-efficacy with regard to integrating occupational factors within their practice. However, although these positive findings and the fact that most respondents stated that they involve the occupational factors of their patients within their practice, 64.1% indicated that occupational factors should be addressed to a greater extent. It seems that fulfilling the items mentioned in the I-Change model [28], does not necessarily lead to adequate behavior with regard to addressing occupational factors within generalist physical therapy. Thus, although respondents do address occupational factors, most respondents think that the patient’s work should be more extensively addressed.

With regard to working together with supervisor/colleagues, most respondents indicated that they rarely or only sometimes address this topic. Relationships at work is one important psychosocial factor and bad relationships at work are an important barrier for recovery for, e.g., chronic low back pain [29]. This is line with previous qualitative research in which participants emphasized that patients need to be questioned about their work, including all work features and psychosocial factors [26]. Another study showed that psychosocial factors are a major barrier for return to work [30]. Also, musculoskeletal PTs are aware of the potential benefits of incorporating psychological interventions within their practice, but feel insufficiently trained to use such interventions and do not sufficiently integrate these psychosocial factors within their practice [31, 32]. However, in the present study, work pressure (also a psychosocial factor) was addressed regularly or always by most respondents.

Addressing the patient’s work/work activities is done by the majority of respondents in all phases of care. However, during physical examination, 38.1% of the respondents stated that they never, rarely, or only sometimes pay attention to the work of their patients. Respondents indicated that they give advice to their patients about their workplace, and most respondents let the patient imitate/show their work activities. However, the majority of respondents do not use pictures/videos, nor do they visit the workplace, and 67.4% never or only rarely uses a work-related questionnaire. This indicates that the advice given by generalist PTs is mainly general advice and not based on the specific workplace/work situation of the patient.

### Lack of knowledge and needs of respondents

In previous qualitative research in the Netherlands [26], lack of knowledge of generalist PTs was seen as a barrier for addressing work within PT practice. Participants felt particularly uncertain about what kind of advice they are ‘allowed’ to give patients, and about their knowledge related to workplace laws and regulations. Other topics requiring attention were: knowledge about work-related factors, how to address work-related factors, the workplace, workplace investigations, and knowledge about occupational healthcare providers [26]. In the present study, only 41.6% of the respondents agree or totally agree that, based on their current knowledge, they are optimally able to treat patients who work and to advise them about occupational factors. Of all respondents, 36.6% mentioned that they need more knowledge about work tasks/work activities, work methods/technique, workplace, working hours, and workload. Although most respondents indicated that they were able to address the patient’s work despite the ever-changing laws and regulations, half of the respondents indicated ‘more knowledge about occupational health-related laws and regulations’ as one of their needs.

About 36% of the respondents stated that they need more work-specific knowledge and skills to carry out a basic workplace assessment. Since 60.6% of the respondents indicated that they have a need for the possibility to bill for a workplace assessment, financial considerations might be an additional reason for the low percentage of workplace visits. However, in the Netherlands, from 2017 onwards several insurance companies provide billing opportunities for workplace visits, which meets the need of the respondents. Therefore, upgrading the knowledge and skills of generalist PTs who will carry out these workplace assessments seems necessary.

Other needs of over 50% of the respondents were: questionnaires about work participation and screening lists to assess to what extent the patient’s complaint is work related (this was also an outcome of the previous qualitative research) [26]. Almost 40% of the respondents stated that attention to occupational factors should play a greater role in the basic physical therapy educational programs and in physical therapy guidelines.

### Cooperation with occupational healthcare providers

Our earlier qualitative study revealed that a major issue to be addressed is the lack of cooperation between the PT and (other) occupational healthcare providers, especially between the generalist PT and the WPT/OPT and the occupational health physician [26]. Moreover, the difference between generalist physical therapy and work-related physical therapy/occupational physical therapy was unclear. It was emphasized by the participating PTs and WPTs/OPTs that WPTs/OPTs have specific knowledge and that WPT/OPT is a valuable specialization. However, some respondents mentioned that they were able to address most of the work-related factors themselves [26]. In the present survey, for most of the respondents the difference in competences and responsibilities between generalist PT and OPT was unclear but 77.5% of the respondents do see a role for themselves within the domain of ‘work’. However, for at least part of the respondents, the specific knowledge that is required in the field occupational health is not sufficiently accessible to the generalist PT. Also, more than 40% of the respondents also stated that they need more knowledge about the domain and position of WPTs and other occupational health professionals.

Generalist PTs do refer patients to an occupational health professional. However, this referral process could be improved, especially after the patient interview and in case of a disappointing recovery. Another question is: to which occupational health professional should patients be referred to. It seems that generalist PTs mostly communicate about the patient with the occupational health physician/insurance physician and the occupational therapist. Only 14.8% of the respondents indicated that they communicate with or consult a WPT. If generalist PTs have a colleague WPT within their practice they often do work together. However, if no WPT is present within their practice, only 12.7% of the PTs sometimes/regularly refer patients to a WPT. Only 35.9% of the respondents answered that they had a need for more intensive cooperation with the WPT, whereas 46.5% had a need for a more intensive cooperation with the OPT. Based on the results of qualitative research it was concluded that the cooperation/networking between PTs and WPTs, and knowledge about the WPT, should be enhanced [26]. However, based on the present survey, it seems that although there is little cooperation between generalist PTs and OPTs/WPTs, generalist PTs do not feel the need to work more closely together. Moreover, since almost 50% of the respondents indicated that they have sufficient knowledge of the domain of the WPT to refer patients, they do not see a need for cooperation or have insufficient insight into the additional value of the WPT. In the earlier qualitative study [26], the interaction between participants (WPTs, OPTs and PTs) during the focus group meetings might have contributed to a better perspective with regard to cooperation with the generalist PT and the OPT/WPT.

### Strengths and limitations

Although some studies have evaluated the experiences/perspectives of PTs with regard to RTW [19, 30] or managing a condition in the context of work [33], to our knowledge this is the first study to use a survey to investigate how generalist PTs currently integrate work participation within their practice, and to evaluate their opinions/needs with regard to enhancing the integration of the patient’s work within physical therapy. The developed survey was based on our earlier qualitative research [26], which ensured that a variety of clinically important topics was included in the survey. Moreover, this survey provides a triangulation of the results of the previous qualitative study and evaluated the results of the qualitative study on a broader scale in a group of generalist PTs. Thus, the present study contributes to the body of knowledge with regard to addressing occupational factors within physical therapy practice.

This study also has some limitations. Although the use of digital news-mails and social media increased the chance of reaching almost all PTs in the Netherlands, it was still difficult to find respondents for this study. This was also the case in previous qualitative research, probably indicating that the relevance of this topic was judged as low by PTs [26]. Since potential respondents were recruited by news-mails and announcements, this implies that mainly PTs already interested in work/occupation filled in the survey, which could have led to selection bias and influenced our results. Also, in this study, 23.2% of the respondents had a WPT within their practice. From a total of 19,557 PTs in the Netherlands who are a member of the Royal Dutch Society for Physical Therapy, only 169 are registered with the professional Dutch OPT/WPT association. Therefore, in this study, a relatively large number of respondents had a WPT in their practice, which may have led to more knowledge about occupational factors and a more pronounced positive attitude with regard to the WPT.

It is also possible that some respondents gave wished-for answers on some of the questions in the survey. This could be possible in most of the questions, because we assume that the ideal answers are known by the respondents. In addition, respondents can also have insufficient insight in their own knowledge and skills and overestimate their actual knowledge and skills, which could lead to too positive results of the survey.

Due to the varying background, age, gender and specializations of the respondents, it is a representative sample of generalist PTs. However, the 142 respondents represent only a small proportion of the Dutch PTs, which limits the generalizability of the results. Therefore, the results of this study should be interpreted with caution. Nevertheless, we think that this study is a valuable addition to our previous qualitative research, in which 21 generalist PTs and 9 WPTs/OPTs participated in the focus groups [26].

### Recommendations

Although most respondents were aware of the importance of integrating occupational factors and perceived that they addressed occupational factors within their practice, it seems that the patient interview and clinical reasoning process were based on subjective procedures and impressions. It seems that a more systematic approach to obtain insight into occupational factors by means of questionnaires, observations, and workplace visits could increase effectiveness. This was also supported by the respondents, as the majority indicated that occupational factors should be more extensively addressed within physical therapy. Therefore, it seems valuable to support and facilitate generalist PTs in addressing these factors, i.e. by providing more information about work-related factors, laws and regulations, questionnaires and screening lists, and by incorporating work-related factors in guidelines. This can be done by developing (online) courses and integration of these factors within physical therapy educational programs. Moreover, an easy-to-use guideline to address occupational factors could be developed and it seems valuable to provide an overview of all these topics (e.g. via an online toolbox) [26]. Also, facilitating a systematic approach for addressing work participation within PT practice seems valuable. There is also a lack of knowledge about the domain and added value of the WPT and OPT, and a lack of cooperation. Although enhancing the cooperation with the WPT was not a specific need of most of the respondents (only 35.9% mentioned they want more intensive cooperation with a WPT), cooperation between generalist PTs and WPT should be enhanced [17, 26]. To achieve this, an overview or decision tree could be useful. To facilitate cooperation, a role might be played by the professional associations and the individual therapists [26].

It is also recommended to investigate the actual situation in practice by observations or investigation of the medical files. This would allow to explore areas in which generalist PTs might be unaware of missing competences and knowledge. In addition, it would be interesting to examine how generalist PTs in other countries integrate occupational factors within their practice.

## Conclusions

This study provides insight into how generalist PTs in the Netherlands currently integrate occupational factors within their practice and also identified their opinions/needs with regard to enhancing the integration of the patient’s work within physical therapy practice. Most generalist PTs address occupational factors within their care. However, it seems that occupational factors should me more extensively addressed within physical therapy practice. Many respondents reported that they need more information about work-related factors, laws and regulations, questionnaires, and screening lists. Another need was to have the possibility to bill for a workplace assessment, as well as more knowledge on the domain/position of WPTs and other occupational health professionals. This study also identified a lack of cooperation between generalist PTs and WPTs. The results of this survey can be used to facilitate the integration of occupational factors within physical therapy practice and to enhance cooperation between generalist PTs and WPTs.

## Additional file

Additional file 1: Appendix 1. Overview of the identified main categories and underlying themes in the previous conducted qualitative study [26]. (DOCX 53 kb)

## Abbreviations

MSDs: Musculoskeletal disorders
OPT: Occupational physical therapist
OPTs: Occupational physical therapists
PT: Physical therapist
PTs: Physical therapists
RTW: Return to work
SD: Standard deviations
WPT: Work-related physical therapist
WPTs: Work-related physical therapists
