# Supplementary material for: Work participation of patients with musculoskeletal disorders: is this addressed in physical therapy practice?
Source: J Occup Med Toxicol. 2017 Aug 25;12:27. doi: 10.1186/s12995-017-0174-5 (PMC5574154; doi:10.1186/s12995-017-0174-5)
Supplement: Additional file 1: — Appendix 1. Overview of the identified main categories and underlying themes in the previous conducted qualitative study [26]. (DOCX 53 kb) [file 12995_2017_174_MOESM1_ESM.docx]

Appendix 1: Overview of the identified main categories and underlying themes in the previous conducted qualitative study [26].
